# Genetic Diversity of the KIR/HLA System and Susceptibility to Hepatitis C Virus-Related Diseases

**DOI:** 10.1371/journal.pone.0117420

**Published:** 2015-02-20

**Authors:** Valli De Re, Laura Caggiari, Mariangela De Zorzi, Ombretta Repetto, Anna Linda Zignego, Francesco Izzo, Maria Lina Tornesello, Franco Maria Buonaguro, Alessandra Mangia, Domenico Sansonno, Vito Racanelli, Salvatore De Vita, Pietro Pioltelli, Emanuela Vaccher, Massimiliano Beretta, Cesare Mazzaro, Massimo Libra, Andrea Gini, Antonella Zucchetto, Renato Cannizzaro, Paolo De Paoli

**Affiliations:** 1 Facility Bio-proteomica/Dir. Sc, CRO National Cancer Institute, Aviano, Pordenone, Italy; 2 Experimental and Clinical Medicine, University of Florence, Florence, Italy; 3 Hepatobiliary Unit, National Cancer Institute “Fondazione Pascale”, Naples, Italy; 4 Molecular Biology and Viral Oncology, National Cancer Institute “Fondazione Pascale”, Naples, Italy; 5 Liver, IRCCS Casa Sollievo della Sofferenza Hospital, San Giovanni Rotondo, Italy; 6 Biomedical Sciences and Human Oncology, University of Bari Medical School, Bari, Italy; 7 Medical and Biological Sciences, University Hospital Santa Maria della Misericordia, Udine, Italy; 8 Hematology and Transplant Unit, San Gerardo Hospital, University of Milano-Bicocca, Monza, Italy; 9 Medical Oncology, Centro di riferimento oncologico, Aviano, Pordenone, Italy; 10 Biomedical Sciences, University of Catania, Catania, Italy; 11 Epidemiology and Biostatistics, CRO National Cancer Institute, Aviano, Pordenone, Italy; 12 Gastroenterology, CRO National Cancer Institute, Aviano, Pordenone, Italy; University of Sydney, AUSTRALIA

## Abstract

**Background:**

The variability in the association of host innate immune response to Hepatitis C virus (HCV) infection requires ruling out the possible role of host KIR and HLA genotypes in HCV-related disorders: therefore, we therefore explored the relationships between KIR/HLA genotypes and chronic HCV infection (CHC) as they relate to the risk of HCV-related hepatocarcinoma (HCC) or lymphoproliferative disease progression.

**Methods and Findings:**

We analyzed data from 396 HCV-positive patients with CHC (n = 125), HCC (118), and lymphoproliferative diseases (153), and 501 HCV-negative patients. All were HIV and HBV negative. KIR-SSO was used to determine the KIR typing. KIR2DL5 and KIR2DS4 variants were performed using PCR and GeneScan analysis. HLA/class-I genotyping was performed using PCR-sequence-based typing. The interaction between the KIR gene and ligand HLA molecules was investigated. Differences in frequencies were estimated using Fisher’s exact test, and Cochran-Armitage trend test. The non-random association of KIR alleles was estimated using the linkage disequilibrium test. We found an association of KIR2DS2/KIR2DL2 genes, with the HCV-related lymphoproliferative disorders. Furthermore, individuals with a HLA-Bw6 KIR3DL1+ combination of genes showed higher risk of developing lymphoma than cryoglobulinemia. KIR2DS3 gene was found to be the principal gene associated with chronic HCV infection, while a reduction of HLA-Bw4 + KIR3DS1+ was associated with an increased risk of developing HCC.

**Conclusions:**

Our data highlight a role of the innate-system in developing HCV-related disorders and specifically KIR2DS3 and KIR2D genes demonstrated an ability to direct HCV disease progression, and mainly towards lymphoproliferative disorders. Moreover the determination of KIR3D/HLA combination of genes direct the HCV progression towards a lymphoma rather than an hepatic disease. In this contest IFN-α therapy, a standard therapy for HCV-infection and lymphoproliferative diseases, known to be able to transiently enhance the cytotoxicity of NK-cells support the role of NK cells to counterstain HCV-related and lymphoproliferative diseases.

## Introduction

The World Health Organization (WHO) estimates that about 3% of the world population is infected with the Hepatitis C virus (HCV), and three to four million individuals are newly infected each year. Although new antiviral treatments are very promising [1], today only a minority of patients successfully clear up HCV infections, and the remaining patients (60–85%) develop chronic infection.

The patients with persistent infection are at risk of developing chronic liver lesions, ranging from minimal inflammation to cirrhosis and hepatocellular carcinoma (HCC). Moreover, chronic infections could cause several extrahepatic diseases, including the type II mixed cryoglobulinemia (MC), a systemic autoimmune disease characterized by a monoclonal/oligoclonal proliferation of B-cells. This produces immune complex-mediated disorders and may evolve in a small fraction of patients to frank malignant B-cell non-Hodgkin’s lymphoma (NHL) [2].

The mechanisms whereby HCV establishes chronic infection, autoimmune diseases, HCC or NHL are still poorly understood. Viral factors and determinants of the host are both involved in HCV clearance and disease pathogenesis. Spontaneous viral clearance has been associated with a low viral diversity and strong CD8+ and CD4+ T-cell responses targeting a broad range of viral epitopes [3]. At the same time, infectious agents are known to develop a high variety of mechanisms, which finally lead to an ineffectual T-cell response and a persistent infection. Currently, the exact mechanism responsible for the T-cell failure of HCV infection is not well-defined, although several evidences suggest that HCV mutations within human leukocyte antigen (HLA)-restricted epitopes, which are no longer recognized by T-cells and neutralizing antibodies, are one of the most potent strategies utilized by HCV. Moreover, other mechanisms potentially contributing to virus persistence include the HCV interference with host cellular components and signaling pathways [4]. Additionally, alcohol consumption, age, sex and genetics cofactors are also important predisposing conditions.

Natural killer (NK) cells are part of the innate immune system that plays an important role in HCV infection by killing infected/altered cells through a direct and antibody-dependent cell-mediated (ADCC) cytotoxic action and through the release of cytokines that activate and go back other effector cells of the immune system [5;6].

NK cell activity is mainly regulated by NK receptors, which include the killer cell Ig like receptors (KIR), encoded by a family of activating and inhibitory genes located on the human chromosome 19. At present, a total of 16 KIR human genes have been characterized, of which 8 are NK-cell inhibitors (KIR2DL1–5, 3DL1–3), 6 are activators (KIR2DS1–5, 3DS1) and 2 are pseudo genes (KIR2DP1, 3DP1). Contrary to T-cell, which recognize tumor antigens presented by HLA molecules, NK cells can also recognize the “loss” of HLA induced by transformation, cellular stress or infection. Different levels of NK-cell activation are modulated by various KIR interactions with their cognate HLA ligand, resulting in an overall balance of signals from activating versus inhibitory receptors. Depending on both the inheritance of stochastic KIR and HLA genes, as well as levels of KIR/HLA surface expression levels, a cell tolerance or rejection occurs [7].

NK cells have also been reported to play a role in HCV clearance [5]. In particular, NK cells were demonstrated to mediate the inhibition of HCV-replication and to exert a targeted cytotoxic action against targeted cells given that NK cells isolated from healthy donors kill HCV-replicating cells and secrete IFN-γ [8;9]. However, there are evidences that HCV-mediated interferences with the action of NK cells [10;11].

HLA-C1 KIR2DL3^+^ in homozygosis has been associated with HCV clearance in several studies [12;13] but the occurrence of this association was not always observed. At the same time, HLA-C1 KIR2DL3^+^ has also been associated with sustained virologic response to anti-HCV therapy [10;11]. Moreover, a protective role for HLA-Bw4 KIR3DS1^+^ against liver disease progression has been proposed [14;15].

Liver NK cells are abundant in the liver and play beneficial roles in inhibiting viral infection, tumor cell growth and liver fibrosis, but they can also play detrimental roles in stimulating liver injury and attenuating liver regeneration by directly killing target cells and the production of IFN-γ. Conversely, inhibition of NK cells may play a defensive role by protecting liver cells from inflammation and injury, as supported by some experimental models [16–18]. Both the frequency of liver and peripheral NK cells and their function were found to decrease in HCC patients, suggesting a potential protective role, although many aspects of their exact involvement in HCC still needs to be deciphered [5;19–22]. The purpose of this study is to examine KIR genotypes and HLA KIR compounds in chronic HCV-infected patients with HCC and malignant extrahepatic lymphoproliferative diseases in order to investigate the potential effect of the host HLA/KIR profile on HCV-related disease progression.

## Methods

### Ethics Statement

CRO Aviano National Cancer Institute Review Board (IRB) have approved the study. Ethical principles espresse in the Declaration of Helsinki has been respected. Informed written or oral consent have been obtained from the participants as reported in the Methods section of the manuscript

### Patient characteristics

A total of 396 HCV-infected (HCV-positive) and 501 non HCV-infected (HCV-negative) Italian patients were selected for the present study. Patients were recruited from 9 Italian centers (CRO Aviano National Cancer Institute, Pordenone, “S. Maria degli Angeli” General Hospital, Pordenone; University of Bari Medical School, Bari; “S. Maria della Misericordia” General hospital, Udine; University of Florence, Florence; “IRCCS Casa Sollievo della Sofferenza,” San Giovanni Rotondo, Bari; National Cancer Institute “Fondazione Pascale,” Naples; Ospedale “S. Gerardo di Monza,” Milan; University of Catania, Catania).

The diagnosis for chronic HCV infection is based essentially on the positivity of antibodies against HCV and serum transaminase (ALT) levels. Test immunoenzimatico (III-generation EIA) against HCV-core and HCV-non structural antigens were used. There are several commercial tests for the determination of the genotypes: the mostly used was the test Inno-Lipa. As regard the diagnosis of HCC, clinical centers used the standard criteria listed in the European Association for the Study of the Liver (EASL) that incorporate both invasive and noninvasive measures [23] Noninvasive criteria include two imaging techniques, both demonstrating a focal lesion >2 cm in diameter with features of arterial hypervascularization. Most coauthors are members of the A.L.CRI (Associazione Italiana per la Lotta alle Crioglobulinemie) an association that proposed and validate the consensus protocol for detection and typing of cryoglobulins [24]. NHL in the course of HCV infection has been confirmed by histopathologists based on WHO classification. [25].

Both groups of patients were tested negative for HBV and HIV infections. Among HCV-positive patients, 125 had a chronic HCV infection but without HCC or any sign/symptom of definite MC or NHL (CHC n = 125), patients with HCV-related hepatocellular carcinoma (HCC n = 118), and patients with a lymphoproliferative disease (n = 153) including a group affected by either a definite cryoglobulinemic syndrome according to previously described criteria [23] (MC n = 75) or a definite malignant B-cell non-Hodgkin’s lymphoma (NHL n = 78). Our reference series of HCV negative cohort members included 501 individuals with negative virological tests for HCV, HBV or HIV with no clinical evidence of neoplastic or autoimmune disorders (n = 79), with a celiac disorder (n = 76) or with a gastric /intestinal neoplasia (n = 346). Informations concerning the demographic and clinical characteristics of the patient’s groups were reported on Table 1.

**Table 1:** Demographics features and HCV genotypes in HCV-negative and in patients with HCV infections and different outcomes.

	HCV negative No. (%)	HCV-positive patients		
		Chronic HCV infection No. (%)	Hepatocellular carcinoma No. (%)	Lymphoproliferative disease No. (%)
No. patients	501 (100.0)	125 (100.0)	118 (100.0)	153 (100.0)
				
Age				
Mean (± SD)	56.2 (± 13.3)	56.2 (± 13.3)	67.1 (± 10.2)	60.8 (± 10.4)
				
Gender				
Male	311 (62.1)	60 (48.0)	75 (63.6)	58 (37.9)
Female	190 (37.9)	65 (52.0)	43 (36.4)	95 (62.1)
				
HCV genotypes				
1	-	59 (47.2)	57 (48.3)	91 (59.5)
2	-	27 (21.6)	17 (14.4)	43 (28.1)
3–4	-	14 (11.2)	4 (3.4)	8 (5.2)
Nd	-	25 (20.0)	40 (33.9)	11 (7.2)
Negative	501 (100.0)	-	-	-

Nd: HCV genotype not determined

Genomic DNA was extracted from peripheral blood sample using the EZ1 DNA blood kit and the BioRobot EZ1 Workstation (Qiagen Inc., Valencia, CA).

Samples were collected after obtaining informed written consent.

.

### PCR-SSP KIR typing

All the KIR/HLA typing has been performed in a unique center (CRO). Genomic DNA was used to determine the genotype of KIR genes using Lifecodes KIR-SSO typing kit for use with Luminex (Gen-Probe Transplant Diagnostic, GTI for Italy) according to the manufacturer’s instructions. The presence or absence of the following 16 *KIR* genes was identified: *KIR2DL1*, *KIR2DL2*, *KIR2DL3*, *KIR2DL4*, *KIR2DL5*, *KIR2DS1*, *KIR2DS2*, *KIR2DS3*, *KIR2DS4*, *KIR2DS5*, *KIR3DL1*, *KIR3DL2*, *KIR3DL3*, *KIR3DS1*, *KIR2DP1* and *KIR3DP1*.

KIR gene profiles were determined by the presence or absence of each KIR gene in a given individual. This method of KIR typing does not allow the direct determination of KIR2DL2 copy number. Instead, we used the allelic nature of KIR2DL2 and KIR2DL3 at the 2DL2/2DL3 locus to infer the number of copies of KIR2DL2.

KIR2DL5 A and B subtyping was performed using polymerase chain reaction (PCR), as we previously reported [24]. KIR2DS4 was typed for encoded cell-surface receptor (full) or a truncated protein variant with loss of the transmembrane and cytoplasmatic domains (del) by GeneScan analysis with a 6-FAM labeled primer [24]. The deleted variant of KIR2DS4 was not anchored to the cell membrane but was encoded for a soluble form of the protein that is potentially secreted and likely lacks function(s).

### KIR genotype assessment

KIR genotype was assigned according to the database (http://www.allelefrequencies.net/). All genotype contained KIR2DL4, KIR3DL2, KIR3DL3, KIR2DP1 and KIR3DP1 framework genes. In addition, genotypes were assumed to contain either 2DL5A or 2DL5B, and 2DS4full or 2DS4del.

In the assessment of the KIR genotype, group B genotypes were defined by the presence of one or more of the following genes: KIR2DL5, KIR2DS1, KIR2DS2, KIR2DS3, KIR2DS5 and KIR3DS1. Conversely, stable group A genotype was defined by the absence of all these genes and by the presence of KIR3DL1, KIR2DL1, KIR2DL3 and KIR2DS4 genes. A comparison was done with the frequencies reported in the database for the Caucasian population [25] and linkage disequilibrium organization of the complex human KIR superlocus [26].

**Fig 1:**
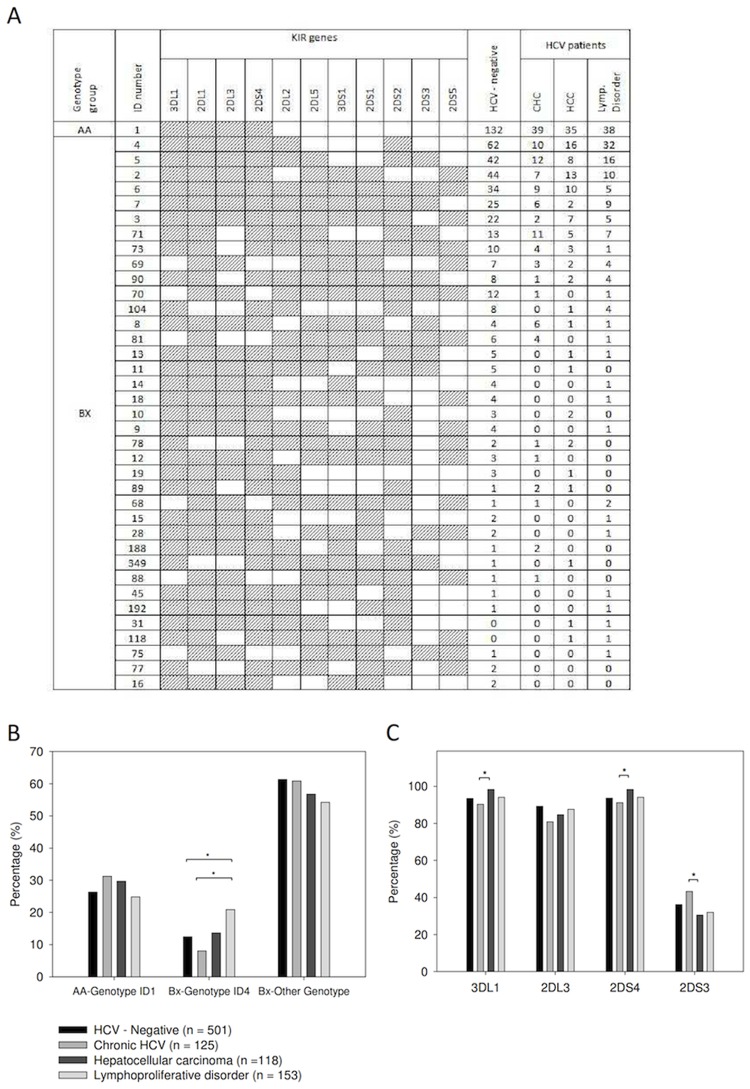
KIR gene profiles in our study populations. A total of 42 KIR gene profiles were identified (panel A). Genotype ID number reported are those from the reference database [28]. The presence of KIR genes is indicated by grey shanding. Genotypes AA and BX according to criteria reported in material and method section are indicated in the first column.°Only ID Genotypes present in at least two patients were reported in tables. Distribution of genotype ID1 and ID4 for HCV-negative patients and for patients with HCV infection and different outcomes (panel B). Distribution of KIR3DL1, KIR2DL3, KIR2DS4, KIR2DS3 genes ID4 for HCV-negative patients and for patients with HCV infection and different outcomes (panel C). * Fisher’s Exact test, p<0.05.

Moreover, centromeric (Cent) and telomeric (Tel) regions splits in half the KIR genotype; KIR3DL3 and KIR3DP1 delimited the centromeric part of the KIR locus, whereas KIR2DL4 and KIR3DL2 delimited the telomeric part (Fig. 1). KIR2DL5, KIR2DS5 and KIR2DS3 genes can be present both in centromeric and telomeric locations.

### SBT HLA typing

HLA genotyping was performed using PCR-sequence-based typing (PCR-SBT) with primers specific for each class I locus (A, B and C) [24].

Two novel HLA class I alleles (A*02:374 and B*51:141) were identified in this study and submitted in the GenBank nucleotide sequence database.

### KIR/HLA interactions

Several interactions between KIR genes and HLA ligands were contemplated. HLA-C molecules, the KIR-ligands, were classified as either HLA-C1 or HLA-C2 on the basis of dimorphisms at position 80. So far, known KIR-HLA pairs are HLA-C2 epitope (Asparagine at position 77, Lysine at position 80) 2DL1/2DS1^+^ and HLA-C1 epitope (Serine at position 77, Asparagine at position 80) 2DL2/3/2DS2^+^, HLA-Bw4 3DL1/3DS1^+^, HLA-C*04 2DS4^+^ and HLA-A3/A11 3DL2^+^.

HLA-Bw4–80I (Isoleucine at position 80) was recognized to bind KIR3DL1 with higher affinity than Bw4–80T (Treonine at position 80), so we also analyzed the frequencies of Bw4-containing allotypes based on their dimorphism at position 80 [30].

Ligands for the lasting KIRs remain elusive.

### Statistical analysis

The genotypes of KIR gene and KIR-HLA pair frequencies were individually determined by direct counting of the individual who tested positive for a specific (pair of) gene. Linkage disequilibrium (LD) between pairwise KIR genes was assessed with Cramer’s V correlation coefficient. Differences in frequencies were estimated by using Fisher’s exact test and trend in frequencies were analyzed with Cochran-Armitage trend test (z^2^). A p-value of <0.05 was considered significant.

## Results

### KIR genotypes and single KIR genes

In this study, we found 65 different KIR genotype profiles: the 38 that were present in at least two patients are reported in Fig. 1A. The framework of KIR2DL4, KIR3DL2, KIR3DL3 and KIR3DP1 genes were present in all individuals; thus, they are not reported in the Figure. Two new genotypes, indicated as “*new”* in the identification number column (ID), were found and reported in the S1 Table.

The genotype ID 4 frequency in patients with CHC was found to be lower than in HCV-positive patients with lymphoproliferative disorder (8% *versus* 20.9%, Fisher’s exact test, p<0.01, Fig. 1B). Furthermore, the genotype ID 4 frequency in patients with lymphoproliferative disorder was higher than in HCV-negative patients (20.9% *versus* 12.4%, p = 0.01, Fig. 1B) and higher than that found in the reference Caucasoid ethnicity (ranging between 18.0% and 6.0% in Portugal and in Iran East Azezebaijan, respectively) [25] suggesting that the genotype ID 4 is in relation with HCV-related lymphoproliferative disorders, particularly NHL.

**Fig 2:**
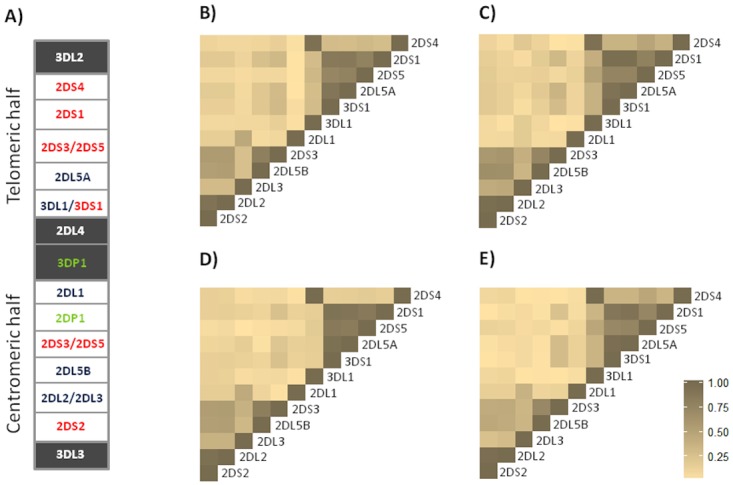
Centromeric and telomeric halves of KIR genotypes (panel A). A stretch of 14 kb DNA that interconnects KIR3DP1 and KIR2DL4 divides the KIR genotype into two halves. The centromeric half is delimited by 3DL3 and 3DP1, while the telomeric half is delimited by 2DL4 and 3DL2. There is different KIR gene content, due to a recombination of these genes, in KIR genotypes across individuals and populations. The framework genes, present in all genotypes are shown in grey boxes; genes encoding activating KIR are in red color; and those for inhibitory receptors are in blue color. KIR2DL4 encodes a receptor that has both inhibitory and activating functions The KIR2DP1 and 3DP1 (green) are pseudogenes that do not express a receptor. **Pairwise D’ LD based on Cramer’s V correlation coefficient between the presence and absence of different KIR genes in four groups of patients (panel B-E)** B: HCV negative; C: Chronic HCV; D: Hepatocellular carcinoma; E: Lymphoproliferative disease. The KIR cluster genetic polymorphism is considered as the presence or absence of KIR genes.

Classic linkage disequilibrium (LD) studies identified two distinct regions in the KIR cluster around the KIR2DL4 gene: a centromeric (cen) region, which seems to be driven by the KIR2DL5 and KIR2DL2/3 loci, and a telomeric (tel) region driven by the KIR3DL1/S1 locus. The overall patterns of LD of KIR receptors were similar in all groups of patients examined in this study (Fig. 2). The genotype ID4 found to be associated with HCV-lymphoproliferative disorders is distinguished from the most prevalent ID 1 for the presence of the KIR2DS2 in strong LD with the KIR2DL2 gene.

When the frequency of single KIR genes was compared a number of statistically significant differences in HCV-positive groups (Fig. 1C) was identified. Among these genes, the frequency of KIR2DS3 gene was lower in HCC malignancies than in CHC (30.5% in HCC *versus* 43.2% in CHC, p = 0.05, Fig. 1C). Moreover, although the differences in HCV-negative groups did not reach a statistical significance, the frequency of this gene was found to be lower in HCV-negative individuals (36.1%) than in CHC patients (43.2%). The possibility of inheriting both an absence of the KIR2DS3 gene and KIR2DS2/KIR2DL2 genes is about 47–48% in the worldwide population, and this data underlines the specific KIR2DS3 association with CHC [28].

To contrast the findings for the KIR2DS3 that indicated the frequency was higher in patients with CHC, both KIR3DL1 and KIR2DS4 gene frequencies were lower in CHC than in HCC cases (Fig. 1C). Both of these genes are in strong LD (Fig. 2), and according to LD they are rarely inherited (≤25%) along with the KIR2DS3 gene.

### Centromeric and Telomeric KIR locus

Subdividing the KIR cluster into the cent and tel regions identified by the LD study, simplifies the description of the haplotype structure of the KIR genes. This case series (Table 2) found 9 different centromeric gene motifs (Cent 1–9), 8 telomeric gene motifs (Tel 1–8) and 6 centromeric/telomeric gene (Cent/Tel 1–6) motifs.

**Table 2:** Distribution of centromeric/telomeric KIR regions, based on the KIR genotype (Fig. 1), in HCV-negative and in patients with HCV infection and different outcomes.

ID	**KIR GENES**				Blood Donors (HCV-neg) No. (%)	HCV-pos Patients		
	**2DS2**	**2DL2**	**2DL3**	**2DL1**		Chronic HCV No. (%)	Hepatocellular carcinoma No. (%)	Lymphoproliferative disorders No. (%)
Cent1			X	X	203 (40.5)	56 (44.8)	51 (43.2)	57 (37.2)
Cent2	X	X	X	X	222 (44.3)	43 (34.4)	46 (39.0)	75 (49.0)
Cent3		X	X	X	9 (1.8)	0 (0.0)	1 (0.8)	1 (0.6)
Cent4	X		X	X	9 (1.8)	2 (1.6)	2 (1.7)	0 (0.0)
Cent5		X		X	0 (0.0)	0 (0.0)	0 (0.0)	1 (0.6)
Cent6	X	X		X	38 (7.6)	23 (18.4)	14 (11.9)	14 (9.1)
Cent7	X	X			16 (3.2)	1 (0.8)	4 (3.4)	4 (2.6)
Cent8	X	X	X		3 (0.6)	0 (0.0)	0 (0.0)	1 (0.6)
Cent9			X		1 (0.2)	0 (0.0)	0 (0.0)	0 (0.0)
Total					501 (100.0)	125 (100.0)	118 (100.0)	153 (100.0)
ID	**3DS1**	**2DS1**	**3DL1**	**2DS4**				
Tel1			X	X	270 (53.9)	74 (59.2)	70 (59.3)	98 (64.0)
Tel2	X	X	X	X	163 (32.5)	37 (29.6)	42 (35.6)	39 (25.5)
Tel3		X	X	X	18 (3.6)	0 (0.0)	2 (1.7)	4 (2.6)
Tel4	X		X	X	16 (3.2)	2 (1.6)	2 (1.7)	3 (2.0)
Tel5	X	X		X	1 (0.2)	1 (0.8)	0 (0.0)	0 (0.0)
Tel6	X	X			31 (6.2)	11 (8.8)	2 (1.7)	9 (5.9)
Tel7				X	1 (0.2)	0 (0.0)	0 (0.0)	0 (0.0)
Tel8			X		1 (0.2)	0 (0.0)	0 (0.0)	0 (0.0)
Total					501 (100.0)	125 (100.0)	118 (100.0)	153 (100.0)
ID		**2DL5**	**2DS3**	**2DS5**				
Cent/Tel1		X	X	X	69 (13.8)	18 (14.4)	13 (11.0)	10 (6.5)
Cent/Tel2		X		X	94 (18.7)	18 (14.4)	25 (21.2)	24 (15.7)
Cent/Tel3		X	X		110 (22.0)	36 (28.8)	23 (19.5)	39 (25.5)
Cent/Tel4			X		2 (0.4)	0 (0.0)	0 (0.0)	0 (0.0)
Cent/Tel5		X			3 (0.6)	0 (0.0)	1 (0.8)	2 (1.3)
Cent/Tel6					223 (44.5)	53 (42.4)	56 (47.5)	78 (51.0)
Total					501 (100.0)	125 (100.0)	118 (100.0)	153 (100.0)

The total KIR gene profiles are grouped in 9 different centromeric locus (Cent 1–9), 8 telomeric locus (Tel 1–8) and 6 Cent/Tel locus (Cent/tel 1–6), which include genes that can be present in both the centromeric or the telomeric region. The presence of KIR genes in the single region is indicated by the presence of X symbol.

**Fig 3:**
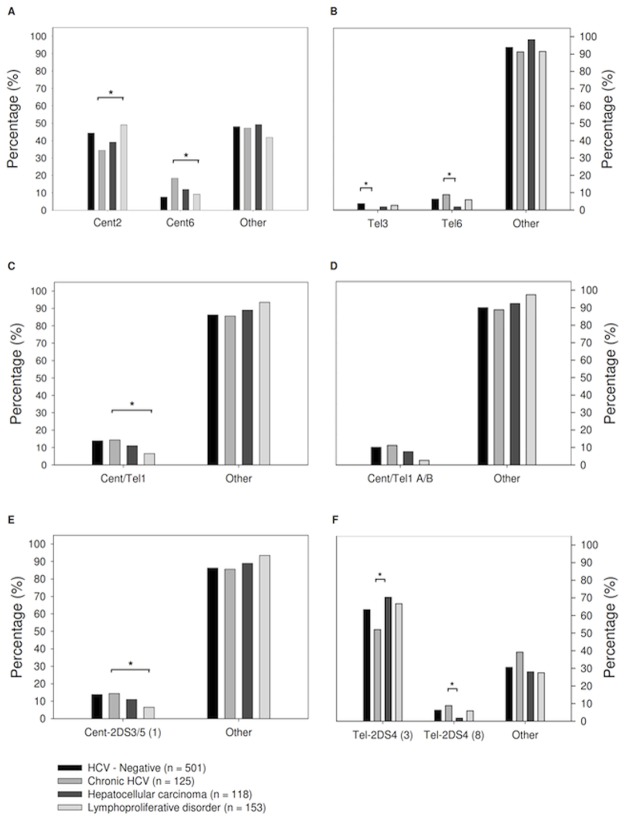
Cent/Tel KIR gene profiles in HCV-negative patients and patients with HCV infection and different outcomes. Distribution of Cent2 and Cent6 locus (panel A, Table 2). Distribution of Tel3 and Tel6 loci (panel B, Table 2). Distribution of Cent/Tel1 loci (panel C, Table 2). Distribution of Cent/Tel1 with A/B KIR2DL5 variant (panel D, Table 3). Distribution of Cent-2DS3/5 (1) loci (panel E, Table 3). Distribution of Tel-2DS4 (3) AND Tel-2DS4 (8) locus (panel F, Table 3). * Fisherx’s Exact test, p<0.05.

With regards to the centromeric KIR regions, the Cent 2 gene-content motif was more frequent in patients with lymphoproliferative disorder than in patients with CHC (49.0% *versus* 34.4%, p = 0.02, Fig. 3A). In contrast, in lymphoproliferative disorder patients, Cent 6 motif was lower than in CHC patients (9.1% versus 18.4%, p = 0.03, Fig. 3A). The Cent2 and Cent6 motifs were present in about 44.3% and 7.6% of the HCV-negative patients. The KIR2DL3 gene distinguishes Cent 2 from Cent 6 motifs and was more frequent in lymphoproliferative disorder compared to CHC: however, this difference did not reach statistical significance (87.6% *versus* 80.8%, Fig. 1C).

**Table 3:** Distribution of Cent/Tel motif and KIR2DL5 A/B and KIR2DS4 Full/del variants in KIR genotype (Fig. 1).

ID	2DL5 (A/B)	**KIR GENES**				Blood Donors (HCV-neg)* No. (%)	HCV-pos Patients		
		**2DL5A**	**2DL5B**	**2DS3**	**2DS5**		Chronic HCV No. (%)	Hepatocellular carcinoma No. (%)	Lymphoproliferative disorders No. (%)
Cent/Tel1	A	X		X	X	15 (3.0)	4 (3.2)	3 (2.5)	6 (3.9)
	B		X	X	X	2 (0.4)	0 (0.0)	1 (0.8)	0 (0.0)
	A/B	X	X	X	X	50 (10.1)	14 (11.2)	9 (7.6)	4 (2.6)
Cent/Tel2	A				X	87 (17.5)	15 (12.0)	24 (20.3)	22 (14.4)
	B		X		X	1 (0.2)	0 (0.0)	1 (0.8)	0 (0.0)
	A/B	X	X		X	6 (1.2)	3 (2.4)	0 (0.0)	2 (1.3)
Cent/Tel3	A	X		X		16 (3.2)	8 (6.4)	4 (3.4)	9 (5.9)
	B		X	X		62 (12.5)	21 (16.8)	16 (13.6)	23 (15.0)
	A/B	X	X	X		29 (5.8)	7 (5.6)	3 (2.5)	7 (4.6)
Cent/Tel5	A	X				3 (0.6)	0 (0.0)	0 (0.0)	1 (0.6)
	B		X			0 (0.0)	0 (0.0)	1 (0.8)	1 (0.6)
Cent/Tel 4	-			X		2 (0.4)	0 (0.0)	0 (0.0)	0 (0.0)
Cent/Tel 6	-					223 (45.0)	53 (42.4)	56 (47.5)	78 (51.0)
Total						496 (100.0)	125 (100.0)	118 (100.0)	153 (100.0)
ID			**2DL1**	**2DS3**	**2DS5**				
Cent-2DS3/5 (1)			X	X	X	69 (13.8)	18 (14.4)	13 (11.0)	10 (6.5)
Cent-2DS3/5 (2)			X		X	87 (17.4)	17 (13.6)	23 (19.5)	24 (15.7)
Cent-2DS3/5 (3)			X	X		109 (21.8)	36 (28.8)	22 (18.6)	39 (25.5)
Cent-2DS3/5 (4)			X			216 (43.1)	53 (42.4)	56 (47.5)	75 (49.0)
Cent-2DS3/5 (5)				X	X	0 (0.0)	0 (0.0)	0 (0.0)	0 (0.0)
Cent-2DS3/5 (6)					X	7 (1.4)	1 (0.8)	2 (1.7)	0 (0.0)
Cent-2DS3/5 (7)				X		3 (0.3)	0 (0.0)	1 (0.8)	0 (0.0)
Cent-2DS3/5 (8)						10 (2.0)	0 (0.0)	1 (0.8)	5 (3.3)
Total						501 (100.0)	125 (100.0)	118 (100.0)	153 (100.0)
ID	2DS4 variant		**3DL1**	**2DS4F**	**2DS4D**				
Tel-2DS4 (1)	Full+Del		X	X	X	109 (21.8)	36 (28.8)	26 (22.0)	28 (18.3)
Tel-2DS4 (2)	Full		X	X		41 (8.2)	12 (9.6)	7 (5.9)	14 (9.1)
Tel-2DS4 (3)	Del		X		X	317 (63.3)	65 (52.0)	83 (70.3)	102 (66.7)
Tel-2DS4 (4)	-		X			1 (0.2)	0 (0.0)	0 (0.0)	0 (0.0)
Tel-2DS4 (5)	Full+Del			X	X	1 (0.2)	0 (0.0)	0 (0.0)	0 (0.0)
Tel-2DS4 (6)	Full			X		0 (0.0)	0 (0.0)	0 (0.0)	0 (0.0)
Tel-2DS4 (7)	Del				X	1 (0.2)	1 (0.8)	0 (0.0)	0 (0.0)
Tel-2DS4 (8)	-					31 (6.2)	11 (8.8)	2 (1.7)	9 (5.9)
Total						501 (100.0)	125 (100.0)	118 (100.0)	153 (100.0)

The Cent/Tel KIR motif with KIR2DL5 A/B variants are grouped in 13 different locus, the Cent KIR motif with KIR2DL1 and KIR2DS3/KIR2DS5 genes are grouped in 8 different locus (Cent-2DS3/5 1–8), and the Tel KIR motif with KIR2DS4 Full/del variant subtypes are grouped in 8 different locus (Tel-2DS4 1–8). The presence of KIR2DS3, KIR2DS5, KIR2DL5 and KIRDS4 variant genes are indicated by the presence of X symbol.

* The sum didn’t up to total because presence of missing values.

Regarding the telomeric KIR motifs, the Tel 3 motif was completely absent in CHC patients, but present in HCV-negative patients (0% *versus* 3.6%, Fisher’s exact test, p = 0.03 Table 2). Tel 3 motif was found to be characterized by the absence of the KIR3DS1 gene.

Tel 6 motif characterized by the absence of both the KIR3DL1 and 2DS4 genes and the presence of the KIR3DS1 and 2DS1 genes was more frequent in CHC patients than in HCC (8.8% *versus* 1.7%, p = 0.02, Fig. 3B). The Tel 6 motif was found in 6.3% of HCV-negative patients.

### Centromeric/telomeric KIR locus

The Cent/Tel1 motif was characterized by the presence of the KIR2DL5, KIR2DS3 and KIR2DS5 genes, and its frequency was lower in lymphoproliferative disorder cases than in CHC cases (6.5% *versus* 14.4%, p = 0.04, Fig. 3C) In HCV-negative patients, it was found to be 13.8%. Among these genes, the KIR2DS3 compared with CHC patients was less expressed in HCC patients (30.5% *versus* 43.2%, p = 0.05, Fig. 1C) and in lymphoproliferative disorder patients (32.0% versus 43.2%) but the difference was not statistical significant.

### Genomic blocks sharing specific sets of alleles in strong linkage disequilibrium

In European-American individuals, linkage disequilibrium was found to be particularly strong between the KIR2DL5-KIR2DS3/KIR2DS5, KIR2DS3/KIR2DS5-KIR2DL1 and KIR3DL1-KIR2DS4 gene pairs [28]. Moreover, the KIR2DL5 gene A and B variants are known to discriminate the centromeric (B) from the telomeric (A) component of the KIR gene locus [29], and two functionally related variants of the KIR2DS4 gene (*full* and *del)* are reported. The study combined all of this information in order to estimate the weight of single gene/variant discrimination. The presence of both 2DL5 A/B variants and the 2DS3 with 2DS5 genes (Cent/Tel1 A/B, Table 3) was less frequent in lymphoproliferative disorders compared to patients with chronic HCV infection (2.6% versus 11.2%, p < 0.01, Fig. 3D). Rather, among HCV-negative patients the frequency of this motif was 10.1%. Furthermore, the presence of both the KIR2DS3 and the KIR2DS5 genes with KIR2DL1 gene (Cent-2DS3/5 (1), Table 3) was lower in lymphoproliferative disorders cases compared to CHC cases (6.5 *versus* 14.4%, p = 0.04, Fig. 3E), and its frequency was about 13.8% in HCV-negative patients.

The KIR3DL1-KIR2DS4 block is part of a conserved genotype termed “ancestral genotype.” The presence of both the KIR3DL1 gene and the deleted form of the KIR2DS4 gene (KIR2DS4 *del* variant, Tel-2DS4 (3), Table 3), which generates a premature stop codon, revealed a frequency higher in HCC cases (70.3%) compared to CHC cases (52.0%, p < 0.01, Fig. 3F). In contrast, the absence of both the KIR3DL1 and KIR2DS4 genes (Tel-2DS4 (8), Table 3) was less frequent in HCC patients than in CHC patients (1.7% *versus* 8.8%, p = 0.02, Fig. 3F). The frequencies of these two motifs in HCV-negative cases were 63.3% and 6.2% respectively (Table 3).

### HLA frequencies

In a previous study, we found a different association between HLA with HCV-related MC and HLA with NHL [33]; to this we analyzed HLA allele frequencies by dividing the group of patients with lymphoproliferative disorders in the benign MC disease and the more malignant NHL disease.

The frequencies of HLA class I ligands (HLA-A, HLA-B and HLA-C) are reported in Table 4 for both groups of HCV-positive patients and HCV-negative individuals.

**Table 4:** Distribution of HLA class I and HLA class I genotype ligands in HCV-negative and in patients with HCV infection and different outcomes.

HLA and HLA genotype ligands		HCV—neg No. (%)	HCV patients				
			Chronic HCV No. (%)	Hepatocellular carcinoma No. (%)	Lymphoproliferative disorder		
					Cryoglobulinemia syndrome No. (%)	Non-Hodgkin lymphoma No. (%)	Total lymphoproliferative diosrders No. (%)
	**Total patients**	501 (100.0)	125 (100.0)	118 (100.0)	75 (100.0)	78 (100.0)	153 (100.0)
**HLA—A**							
A03/A11	At least one allele	127 (25.4)	41 (32.8)	32 (27.1)	11 (14.7)	19 (24.4)	30 (19.6)
	Absent	374 (74.6)	84 (67.2)	86 (72.9)	64 (85.3)	59 (75.6)	123 (80.4)
	P-value^a^		Ref.	ns	-	-	0.01
							
**HLA-B**							
Bw6	At least one allele	410 (81.8)	105 (84.0)	96 (81.4)	55 (73.3)	65 (83.3)	120 (78.4)
	Absent	91 (18.2)	20 (16.0)	22 (18.6)	20 (26.7)	13 (16.7)	33 (21.6)
	P-value^a^		Ref.	ns	-	-	ns
	P-value^b^		ns	ns	Ref.	ns	-
							
Bw4	At least one allele	326 (65.1)	87 (69.6)	82 (69.5)	57 (76.0)	44 (56.4)	101 (66.0)
	Absent	175 (34.9)	38 (30.4)	36 (30.5)	18 (24.0)	34 (43.6)	52 (34.0)
	P-value^a^		Ref.	ns	-	-	ns
	P-value^b^		ns	ns	Ref.	0.02	-
							
Bw6/Bw4	Bw4-Bw4	91 (18.2)	20 (16.0)	22 (18.6)	20 (26.7)	13 (16.7)	33 (21.6)
	Bw6-Bw4	235 (46.9)	67 (53.6)	60 (50.9)	37 (49.3)	31 (39.7)	68 (44.4)
	Bw6-Bw6	175 (34.9)	38 (30.4)	36 (30.5)	18 (24.0)	34 (43.6)	52 (34.0)
	P-value^a^		Ref.	ns	-	-	ns
	P-value^b^		ns	ns	Ref.	0.03	-
							
Bw4–80I	At least one allele	233 (46.5)	62 (49.6)	57 (48.3)	45 (60.0)	25 (32.0)	70 (45.7)
	Absent	268 (53.5)	63 (49.6)	61 (51.7)	30 (40.0)	53 (68.0)	83 (54.3)
	P-value^a^		Ref.	ns	-	-	ns
	P-value^b^		ns	ns	Ref.	<0.01	-
							
Bw4–80T	At least one allele	138 (27.5)	35 (28.0)	32 (27.1)	21 (28.0)	28 (35.9)	49 (32.0)
	Absent	363 (72.5)	90 (72.0)	86 (72.9)	54 (72.0)	50 (64.1)	104 (68.0)
	P-value^a^		Ref.	ns	-	-	ns
	P-value^b^		ns	ns	Ref.	ns	-

^a^ Fisher’s exact test with chronic HCV infection as reference; ^b^ Fisher’s exact test with cryoglobulinemia syndrome as reference.

Compared to all the other groups, the MC group showed a significantly different frequency of HLA genes. The proportion of patients with HLA-A*03 or HLA-A*11 alleles was found to be lower in lymphoproliferative disorders cases than in CHC (19.6% *versus* 32.8%, p < 0.01). The proportion of HCV-negative individuals with theses alleles was also found to be lower (25.4%).

In addition, differences in frequencies were found for the HLA-Bw4 ligand: the proportion of patients with at least one HLA-Bw4 alleles (Bw4–80I or Bw4–80T) was higher in MC cases than in NHL cases (76.0% *versus* 56.4%, p = 0.02). This difference was related to the presence of Bw4–80I variant. In fact, the frequency of patients with at least one Bw4–80I allele was higher in MC patients than in NHL (60.0% *versus* 32.0%, p < 0.01).

### KIR / HLA pair frequencies

**Fig 4:**
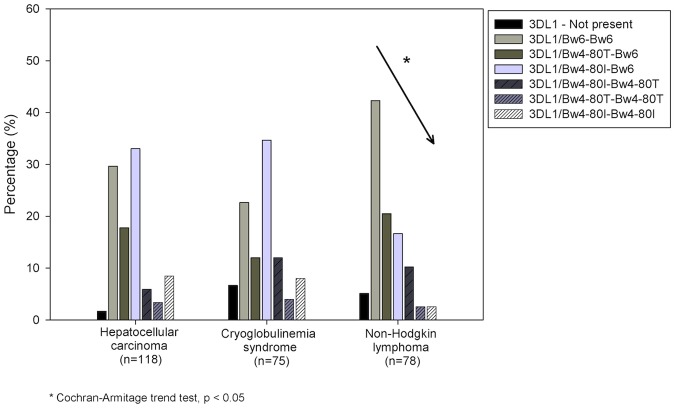
Distribution of KIR3DL1 gene and combination with HLA-B for patients with different HCV related disease outcomes. It is showed a negative trend association between the KIR3DL1/HLA-Bw4 with the presence of HCV-related lymphoma.

Known KIR gene-HLA ligand combinations were examined. Statistical difference between KIR-HLA pair frequencies are reported in Table 5. The frequency of KIR3DL1 gene was found to be higher in HCC cases than in CHC (98.3% *versus* 90.4%, p = 0.01). Furthermore, the proportion of patients having KIR3DL1 with at least one HLA-Bw4 allele (Bw4–80I or Bw4–80T) was lower in patients with NHL than in HCC and MC patients (55.4% *versus*, 69.8% and 75.7%, p = 0.05 and p = 0.01, respectively). Moreover, a significant negative trend was detected for the 3DL1/HLA-B genotype frequencies in NHL patients (from 42.3% for 3DL1/Bw6-Bw6 to 2.6% for both 3DL1/Bw4-I80-Bw4-I80 and 3DL1/Bw4-T80-Bw4-T80 genotypes, Cochran-Armitage trend test z^2^ = 1.98, p = 0.047) (Fig. 4).

**Table 5:** Distribution of KIR/HLA combinations in HCV-negative and in patients with HCV infection and different outcomes.

KIR gene/ HLA—ligand		HCV—neg No. (%)	HCV patients				
			Chronic HCV No. (%)	Hepatocellular carcinoma No. (%)	Lymphoproliferative disorder		
					Cryoglobulinemia syndrome No. (%)	Non-Hodgkin lymphoma No. (%)	No. (%)
	**Total patients**	501 (100.0)	125 (100.0)	118 (100.0)	75 (100.0)	78 (100.0)	153 (100.0)
							
3DL1	Expressed	468 (93.4)	113 (90.4)	116 (98.3)	70 (93.3)	74 (94.9)	144 (94.1)
	Not expressed	33 (6.6)	12 (9.6)	2 (1.7)	5 (6.7)	4 (5.1)	9 (5.9)
	P-value^a^		Ref.	0.01	-	-	ns
							
3DL1/Bw4	Expressed	307 (65.6)	76 (67.3)	81 (69.8)	53 (75.7)	41 (55.4)	94 (65.3)
	Not expressed	161 (34.4)	37 (32.7)	35 (30.2)	17 (24.3)	33 (44.6)	50 (34.7)
	P-value^a^		Ref.	ns	-	-	ns
	P-value^b^		ns	0.05	0.01	Ref.	-
							
3DL1/Bw4-I80	Expressed	217 (46.4)	54 (47.8)	56 (48.3)	41 (58.6)	23 (31.1)	64 (44.4)
	Not expressed	251 (53.6)	59 (52.2)	60 (51.7)	29 (41.4)	51 (68.9)	80 (55.6)
	P-value^a^		Ref.	ns	-	-	ns
	P-value^b^		0.03	0.02	<0.01	Ref.	-
							
2DS4	Expressed	469 (93.6)	114 (91.2)	116 (98.3)	70 (93.3)	74 (94.9)	144 (94.1)
	Not expressed	32 (6.4)	11 (8.8)	2 (1.7)	5 (6.7)	4 (5.1)	9 (5.9)
	P-value^a^		Ref.	0.02	-	-	ns
							
2DS4/Cw04	Expressed	132 (28.1)	33 (29.0)	35 (30.2)	21 (30.0)	31 (41.9)	52 (36.1)
	Not expressed	337 (71.9)	81 (71.0)	81 (69.8)	49 (70.0)	43 (58.1)	92 (63.9)
	P-value^a^		Ref.	ns	-	-	ns
							
3DL2/A03 or A11	Expressed	127 (25.3)	41 (32.8)	32 (27.1)	11 (14.7)	19 (24.4)	30 (19.6)
	Not expressed	374 (74.6)	84 (67.2)	86 (72.9)	64 (85.3)	59 (75.6)	123 (80.4)
	P-value^a^		Ref.	ns	-	-	0.01
	P-value^a^		Ref.	ns	<0.01	ns	-

^a^ Fisher’s exact test with chronic HCV infection as reference; ^b^ Fisher’s exact test with Non-Hodgkin lymphoma as reference;

The frequency of KIR2DS4 was found to be higher in HCC patients compared to CHC patients (98.3% *versus* 91.2%, p = 0.02) but independent from the presence of the HLA-Cw04 ligand (Table 5).

The frequency of the KIR3DL2 gene combining with either HLA-A03+ or HLA-A11+ ligands was higher in CHC patients than in MC patients (32.8% *versus*, 14.7% p < 0.01) (Table 5).

**Activator/Inhibitor KIR genes and HLA matched ligands.** Two different patterns of KIR gene and related combinations of HLA ligands werec examinated (Table 6): the inhibitor motif with all of the 2DL3/2DL2/2DL1/3DL1 KIR genes and the activator motif with the 2DS2/2DS1/3DS1KIR genes.

**Table 6:** Distribution of activator and inhibitor KIR genes and related combination with HLA ligands among HCV-negative individuals and patients with HCV infection.

KIR / HLA—ligand pairs		HCV—neg No. (%)	HCV patients		
			Chronic HCV No. (%)	Hepatocellular carcinoma No. (%)	Lymphoproliferative disorder No. (%)
	**Total patients**	501 (100.0)	125 (100.0)	118 (100.0)	153 (100.0)
**Inhibitor KIR**					
2DL3/2DL2/2DL1/3DL1	All expressed	215 (42.9)	41 (32.8)	47 (39.8)	73 (47.7)
	At least one no expressed	286 (57.1)	84 (67.2)	71 (60.2)	80 (52.3)
	P-value^a^		Ref.	ns	0.01
**HLA ligands:**					
HLA-C2	Present	149 (69.3)	29 (70.7)	37 (78.7)	49 (67.1)
	Not present	66 (30.7)	12 (29.3)	10 (21.3)	24 (32.9)
	P-value^a^		Ref.	ns	ns
					
HLA-C1	Present	171 (79.5)	30 (73.2)	34 (72.3)	58 (79.5)
	Not present	44 (20.5)	11 (26.8)	13 (27.7)	15 (20.5)
	P-value^a^		Ref.	ns	ns
					
HLA-Bw4	Present	142 (66.1)	32 (78.1)	30 (63.8)	47 (64.4)
	Not present	73 (33.9)	9 (21.9)	17 (36.8)	26 (35.6)
	P-value^a^		Ref.	ns	ns
**Activator KIR**					
2DS2/2DS1/3DS1	All expressed	130 (25.9)	32 (25.6)	28 (23.7)	29 (19.0)
	At least one no expressed	371 (74.0)	93 (74.4)	90 (76.3)	124 (81.0)
	P-value^a^		Ref.	ns	ns
**HLA ligands:**					
HLA-C2	Present	93 (71.5)	20 (62.5)	18 (64.3)	19 (65.5)
	Not present	37 (28.5)	12 (37.5)	10 (35.7)	10 (34.5)
	P-value^a^		Ref.	ns	ns
					
HLA-C1	Present	100 (76.9)	27 (84.4)	24 (85.7)	23 (79.3)
	Not present	30 (23.1)	5 (15.6)	4 (14.3)	6 (20.7)
	P-value^a^		Ref.	ns	ns
					
HLA-Bw4	Present	93 (71.5)	25 (78.1)	14 (50.0)	17 (58.6)
	Not present	37 (28.5)	7 (21.9)	14 (50.0)	12 (41.4)
	P-value^a^		Ref.	0.03	ns

^a^ Fisher’s exact test with chronic HCV infection as reference.

The frequency of patients having a full set of KIR-inhibitor genes was found to be higher among lymphoproliferative disorders cases than CHC cases (47.7% *versus* 32.8%, Fisher’s exact test, p = 0.01). However, in patients with the full set of KIR-inhibitors the additional presence of HLA ligands (HLA-C1, HLA-C2 and HLA-Bw4) did not show significant differences in frequency. This suggests that the function of the full set of KIR-inhibitors was not related to the HLA ligands.

The frequency of patients with a full activator motif was found to be lower, but not statistically significant, in patients with lymphoproliferative disorders (19.0%) than in others patients (about 24–26%). However, the proportion of patients showing both activator motif and at least one HLA-Bw4 ligand was found to be lower in HCC patients than in CHC patients (50% *versus* 78.1%, p = 0.03).

## Discussion

The main result of our study is to have shown a significant association between KIR/HLA genotypes to direct toward HCV-related lymphoproliferative diseases. By means of a well represented group of HCV-infected patients, this study compared patients developing a chronic infection with those developing either a malignant hepatic disorder (HCC), or a lymphoproliferative disease. Focus was placed on the identification of KIR gene receptors involved in these different HCV-related diseases as their identification may lead to better understanding of HCV-pathogenesis.

Previous studies identified KIR2DS3 [31] and HLA-C1+ KIR2DL3+, especially when in homozygous [12;13;30] as NK cell-associated KIR genes at a higher frequency in patients who resolved infection compared with CHC patients. Even though a statistically significant difference was not found, we found a trend of reduction of KIR2DL3 gene frequency and an increase in KIR2DS3 gene frequency in CHC patients compared to HCV-negative patients (Fig. 1C). However, this trend was not found in HCV-related malignancies (Fig. 1C). Thus, these data highlight that, in patients who are unable to eliminate the virus, KIR genes that are usually beneficial for HCV eradication are conversely associated to malignancies. Our results are in agreement with several reports describing the functional impairment of NK cells in chronically HCV-infected patients and an influence of HCV viral load towards an increased risk of HCC [32–35] and lymphoproliferative disorders [40].

The decreased association of KIR2DL3 with CHC was restricted to the single KIR2DL3 gene since the centromeric region Cent 2 and Cent 6 frequencies (different only for the presence of the KIR2DL3 gene) among HCV-related groups was in contrast (Table 2). Furthermore, results suggest that KIR2DL3 was more related with lymphoproliferative disorders when the KIR2DL2 gene was also present (Cent 2 compared to Cent 1) (Table 2 and Fig. 3A). In a study on ligand-instructed models of NK-cell education, the recognition of HLA by an inhibitory KIR2DL2 receptor was demonstrated to suppress the subsequent expression of a second KIR2DL1 receptor [37]. Based on this, we proposed that NK cell inhibition derived from the presence of both the KIR2DL2 and KIR2DL3 genes may be weaker that those derived from NK having the presence of the KIR2DL1 gene, and suggested that this reduction in NK cell inhibition was associated with lymphoproliferative disorder.

As regard the KIR2DS3 gene found associated with CHC (Fig. 1C), an analysis of the telomeric/centromeric regions showed that the region including both the 2DS3 and the KIR2DS5 genes (Cent/Tel1) was mostly reduced in lymphoproliferative disorders patients, while the motif excluding the KIR2DS5 gene (Cent/Tel3) was more reduced in HCC patients (Table 2, Fig. 3). These data suggest that the presence of the KIR2DS5 gene might have a protective effect against lymphoproliferative progression in KIR2DS3+ individuals. Although it is tempting to speculate a direct role for KIR2DL2, KIR2DL3, KIR2DS3 and KIR2DS5 in HCV-related lymphoproliferations, it is likely that these genes are all surrogate markers of the same KIR genotype found at a higher frequency in HCV-related lymphoproliferations compared to CHC cases (genotype ID number 4, Fig. 1A and B). Furthermore, a pool of specific KIR/HLA interactions is thought to play a key role in determining whether the lymphoproliferative disorders result in MC cases rather than in the more malignant NHL. Indeed, HLA-B gene analysis showed a negative trend between HLA-Bw4 variants in terms of ligand provision for KIR3DL1 receptors in malignant NHL (Table 4, 5). Data also showed that interaction of HLA-Bw4–80I with the inhibitory KIR3DL1 receptor may influence MC development; thus, individuals with an HLA-Bw4–80I+ KIR3DL1+ genotype would have a low risk of developing NHL (Fig. 4). This data is intriguing; it has been demonstrated that a reduction in NK activation results from a decrease in the number of strongest inhibitor KIR/HLA combinations (i.e., HLA-C2 > HLA-C1> HLA-Bw4), which is in turn coupled with a decrease in the number of activator KIR/HLA pairs [38]. Overall, the number of inhibitor KIR genes was found to be higher in lymphoproliferative disorder patients than in other groups (Table 6), and simultaneously lymphoproliferative disorders showed a reduction in NK activation due to a lower frequency of the activator KIR genes KIR2DS3 and KIR2DS5 (Fig. 3). It is thus hypothesized that an overall inhibition of NK cells may facilitate lymphoproliferative development, and that the involvement of KIR3DL1 is mainly associated with NHL. The higher frequency of KIR3DL1/Bw6 combination found in NHL (Fig. 4) suggests that a HLA-Bw6/antigen-derived complex originating during HCV infection might constitute a signal for the inhibitory KIR3DL1 receptor, as previously demonstrated in other viral situations [39–41]. Although further studies are necessary to confirm these hypotheses, it is now known that IFN-α therapy, a standard therapy for HCV-infection and HCV-related lymphoproliferative diseases, transiently enhances cytotoxicity of NK cells and triggers their activation, rendering these cells more effective against both infection and lymphoproliferation [42].

The role of the KIR genotype in HCV-related HCC is not quite so clear. NK cells are highly enriched in the liver and have been demonstrated to be able to eliminate HCV-infected and transformed cells, especially after Interferon-α stimulation [43]. A protective role of NK cells to resolve HCV infection and liver disease progression has also been demonstrated [44]. Moreover, among patients with liver transplantation and the HLA-C1+ KIR2DL3+ genotype, those having a higher number of activating KIR genes showed a minor HCV recurrence and progression of HCV-related disease [45;46]. Thus, overall data indicates that NK cells and the linked KIR genotype might influence not only HCV viral load, but also the risk of progression to HCC. The most reliable model to explain this finding is a weaker inhibition of NK cells, thus a greater NK activator function may lead to a more efficient elimination of the HCV-infected cells, ultimately protecting the liver from disease progression [47]. Our study results support this association: we found: (i) a higher frequency of KIR3DL1 and KIR2DS4 genes, both representative of the AA-genotype characterized by a reduction of activator KIR genes [48] in HCC cases than in CHC cases (Fig. 1C) and (ii) an association of HCC with the presence of KIR3DL1 and deleted nonfunctional variant of the KIR2DS4D gene, the unique activator gene presents in the AA-genotype (Tel-2DS4 (3), Table 3 and Fig. 3F). Furthermore, in our series, a main characteristic of HCC was the frequent absence of HLA-Bw4+ KIR3DS1+ interaction (Table 6). A protective effect of this interaction on both cirrhosis and HCC has been previously reported in several studies [12;14;32]. Our data confirm this association and highlight its role in counterbalancing the inhibitory effect of HLA-Bw4+ KIR3DL1 interaction.

This study, to the authors’ knowledge, is the first report highlighting the potential impact of the immunogenetic background to address the development of HCV-related lymphoproliferative disorders. HCV-related disorders are multifactorial and complex diseases, but our study highlighted that the persistence of HCV infection after antiviral-therapy remains a significant factor associated with the development of HCC and lymphorpoliferative diseases. In this context, the suggestion of a role where the KIR/HLA genetic background is affecting tumor development and clinical response in the set of HCV-infected patients is intriguing and requires further investigations disclosing perspectives for the development of combining NK-based therapies with conventional antitumoral and anti-HCV treatments.

## Supporting Information

S1 Table Rare KIR genotypes. 27 rare genotype were identified and the ID number reported are those from the reference database [28]. The presence of KIR genes is indicated by the presence of X symbol. Genotypes AA and BX according to criteria reported in material and method section are indicated in the first column.
(DOCX)
