# Correction: Genetic Diversity of the KIR/HLA System and Susceptibility to Hepatitis C Virus-Related Diseases

**DOI:** 10.1371/journal.pone.0128849

**Published:** 2015-05-15

**Authors:** Valli De Re, Laura Caggiari, Mariangela De Zorzi, Ombretta Repetto, Anna Linda Zignego, Francesco Izzo, Maria Lina Tornesello, Franco Maria Buonaguro, Alessandra Mangia, Domenico Sansonno, Vito Racanelli, Salvatore De Vita, Pietro Pioltelli, Emanuela Vaccher, Massimiliano Beretta, Cesare Mazzaro, Massimo Libra, Andrea Gini, Antonella Zucchetto, Renato Cannizzaro, Paolo De Paoli

The 15^th^ author’s name is spelled incorrectly. The correct name is Massimiliano Berretta.
