# Supplementary material for: Genetic Diversity of the KIR/HLA System and Susceptibility to Hepatitis C Virus-Related Diseases
Source: PLoS One. 2015 Feb 20;10(2):e0117420. doi: 10.1371/journal.pone.0117420 (PMC4336327; doi:10.1371/journal.pone.0117420)
Supplement: S1 Table — 27 rare genotype were identified and the ID number reported are those from the reference database [28]. The presence of KIR genes is indicated by the presence of X symbol. Genotypes AA and BX according to criteria reported in material and method section are indicated in the first column. (DOCX) [file pone.0117420.s001.docx]

**S1 Table.**

**Rare KIR genotypes**

27 rare genotype were identified and the ID number reported are those from the reference database (ref. Gonzales). The presence of KIR genes is indicated by the presence of X symbol. Genotypes AA and BX according to criteria reported in material and method section are indicated in the first column.

|  |  | KIR Genes | | | | | | | | | | |  | HCV patients | | | |
| --- | --- | --- | --- | --- | --- | --- | --- | --- | --- | --- | --- | --- | --- | --- | --- | --- | --- |
| Genotype  group | Id  number | 3DL1 | 2DL1 | 2DL3 | 2DS4 | 2DL2 | 2DL5 | 3DS1 | 2DS1 | 2DS2 | 2DS3 | 2DS5 | HCV - negative | CHC | HCC | MC | NHL |
| BX | New | X | X |  | X | X | X | X | X |  | X |  | 0 | 0 | 0 | 1 | 0 |
|  | New | X |  | X | X | X | X |  | X | X | X |  | 1 | 0 | 0 | 0 | 0 |
|  | 94 | X | X |  | X | X | X | X |  | X | X |  | 0 | 0 | 1 | 0 | 0 |
|  | 113 | X | X |  | X | X | X |  | X | X | X |  | 0 | 0 | 1 | 0 | 0 |
|  | 391 | X |  | X | X | X |  |  | X | X |  |  | 0 | 0 | 0 | 1 | 0 |
|  | 206 |  | X | X | X | X | X | X | X |  | X | X | 1 | 0 | 0 | 0 | 0 |
|  | 213 | X | X | X |  | X |  |  |  | X |  |  | 1 | 0 | 0 | 0 | 0 |
|  | 433 | X | X | X | X |  | X | X |  |  |  |  | 1 | 0 | 0 | 0 | 0 |
|  | 22 | X | X | X | X | X | X | X |  | X | X | X | 1 | 0 | 0 | 0 | 0 |
|  | 27 | X | X | X | X |  | X | X |  |  | X |  | 1 | 0 | 0 | 0 | 0 |
|  | 189 | X |  |  | X | X | X | X |  | X |  | X | 1 | 0 | 0 | 0 | 0 |
|  | 344 | X |  | X | X |  |  |  |  |  |  |  | 1 | 0 | 0 | 0 | 0 |
|  | 293 | X |  | X | X | X |  |  |  | X |  |  | 1 | 0 | 0 | 0 | 0 |
|  | 159 |  | X | X |  | X | X | X | X | X | X |  | 1 | 0 | 0 | 0 | 0 |
|  | 51 | X | X | X | X | X | X |  |  |  | X |  | 1 | 0 | 0 | 0 | 0 |
|  | 377 |  |  | X |  | X | X | X | X | X |  | X | 1 | 0 | 0 | 0 | 0 |
|  | 56 | X | X | X | X |  | X | X | X | X | X | X | 1 | 0 | 0 | 0 | 0 |
|  | 191 | X | X | X | X |  |  |  |  | X | X |  | 1 | 0 | 0 | 0 | 0 |
|  | 382 | X | X | X | X | X | X |  | X | X | X | X | 1 | 0 | 0 | 0 | 0 |
|  | 30 | X | X | X | X |  | X |  |  |  | X |  | 1 | 0 | 0 | 0 | 0 |
|  | 97 | X |  |  | X | X | X |  | X | X | X |  | 1 | 0 | 0 | 0 | 0 |
|  | 62 | X | X | X | X | X |  |  |  | X | X |  | 1 | 0 | 0 | 0 | 0 |
|  | 159 |  | X | X |  | X | X | X | X | X | X |  | 1 | 0 | 0 | 0 | 0 |
|  | 156 |  | X | X | X |  |  |  |  |  |  |  | 1 | 0 | 0 | 0 | 0 |
|  | 33 | X | X | X | X |  | X | X | X |  |  |  | 1 | 0 | 0 | 0 | 0 |
|  | 433 | X | X | X | X |  | X | X |  |  |  | X | 1 | 0 | 0 | 0 | 0 |
| BB | 350 |  |  |  |  | X | X | X | X | X |  | X | 1 | 0 | 0 | 0 | 0 |
